# High throughput ^13^C-metabolic flux analysis of 3-hydroxypropionic acid producing *Pichia pastoris* reveals limited availability of acetyl-CoA and ATP due to tight control of the glycolytic flux

**DOI:** 10.1186/s12934-023-02123-0

**Published:** 2023-06-29

**Authors:** Albert Fina, Pierre Millard, Joan Albiol, Pau Ferrer, Stephanie Heux

**Affiliations:** 1 Department of Chemical, Biological and Environmental Engineering, Universitat Autònoma de Barcelona, Bellaterra, Catalonia 08193 Spain; 2 TBI, Université de Toulouse, CNRS, INRAE, INSA, Toulouse, 31077 France

**Keywords:** Fluxomics, ^13^C-Metabolic flux analysis, *Pichia pastoris*, *Komagataella phaffii*, High throughput, 3-hydroxypropionic acid, acetyl-CoA

## Abstract

**Background:**

Production of 3-hydroxypropionic acid (3-HP) through the malonyl-CoA pathway has yielded promising results in *Pichia pastoris* (*Komagataella phaffii*), demonstrating the potential of this cell factory to produce this platform chemical and other acetyl-CoA-derived products using glycerol as a carbon source. However, further metabolic engineering of the original *P. pastoris* 3-HP-producing strains resulted in unexpected outcomes, e.g., significantly lower product yield and/or growth rate. To gain an understanding on the metabolic constraints underlying these observations, the fluxome (metabolic flux phenotype) of ten 3-HP-producing *P. pastoris* strains has been characterized using a high throughput ^13^C-metabolic flux analysis platform. Such platform enabled the operation of an optimised workflow to obtain comprehensive maps of the carbon flux distribution in the central carbon metabolism in a parallel-automated manner, thereby accelerating the time-consuming strain characterization step in the design-build-test-learn cycle for metabolic engineering of *P. pastoris*.

**Results:**

We generated detailed maps of the carbon fluxes in the central carbon metabolism of the 3-HP producing strain series, revealing the metabolic consequences of different metabolic engineering strategies aimed at improving NADPH regeneration, enhancing conversion of pyruvate into cytosolic acetyl-CoA, or eliminating by-product (arabitol) formation. Results indicate that the expression of the POS5 NADH kinase leads to a reduction in the fluxes of the pentose phosphate pathway reactions, whereas an increase in the pentose phosphate pathway fluxes was observed when the cytosolic acetyl-CoA synthesis pathway was overexpressed. Results also show that the tight control of the glycolytic flux hampers cell growth due to limited acetyl-CoA biosynthesis. When the cytosolic acetyl-CoA synthesis pathway was overexpressed, the cell growth increased, but the product yield decreased due to higher growth-associated ATP costs. Finally, the six most relevant strains were also cultured at pH 3.5 to assess the effect of a lower pH on their fluxome. Notably, similar metabolic fluxes were observed at pH 3.5 compared to the reference condition at pH 5.

**Conclusions:**

This study shows that existing fluoxomics workflows for high-throughput analyses of metabolic phenotypes can be adapted to investigate *P. pastoris*, providing valuable information on the impact of genetic manipulations on the metabolic phenotype of this yeast. Specifically, our results highlight the metabolic robustness of *P. pastoris*’s central carbon metabolism when genetic modifications are made to increase the availability of NADPH and cytosolic acetyl-CoA. Such knowledge can guide further metabolic engineering of these strains. Moreover, insights into the metabolic adaptation of *P. pastoris* to an acidic pH have also been obtained, showing the capability of the fluoxomics workflow to assess the metabolic impact of environmental changes.

**Supplementary Information:**

The online version contains supplementary material available at 10.1186/s12934-023-02123-0.

## Background

The methylotrophic yeast *Pichia pastoris* (syn. *Komagataella phaffii*) has gained a lot of attention in the recent years due to its increased use in metabolic engineering applications [1–3]. Recent studies have shown the great potential of *P. pastoris* to produce 3-HP [4, 5]. 3-HP is a bulk chemical with a large interest due to its multiple applications. It was listed among the top-value added products to be obtained from biomass by the Department of Energy of the United States [6]. 3-HP can be converted to acrylic acid, which is used to produce superabsorbent plastics, as well as to other chemicals of interest, such as malonic acid or 1,3-propanediol [6, 7].

3-HP production in *P. pastoris* has recently been achieved by heterologously expressing the bi-functional enzyme malonyl-CoA reductase (MCR) from *Chloroflexus aurantiacus* [4]. Further strain optimization based on rational strain engineering resulted in somewhat limited improvement (ca. 50% increase) of product yield [5] compared to similar strategies to increase the production of 3-HP in other yeasts [8, 9]. So far, the highest 3-HP production reported in *P. pastoris* is 37.1 g L^− 1^ of 3-HP at a volumetric productivity of 0.71 g L^− 1^ h^− 1^ in a fed-batch culture using glycerol as a carbon source [5].

One of the advantages of using *P. pastoris* to produce 3-HP is its ability to grow at a low pH. Performing the cultures at an acidic pH allows the organic acids extraction from the fermentation media using non-toxic solvents. This process is simpler, and it generates less waste products than classical downstream processes [10, 11]. Moreover, in situ product recovery systems can be implemented, avoiding reaching toxic levels of 3-HP [12]. However, while the cultivation of *P. pastoris* at pH as low as 3 has been widely studied as a strategy to minimise the activity of some endogenous proteases [13, 14], there are no previous studies on the impact of low pH on the fluxome of *P. pastoris*.

Recent advances in the field of synthetic biology allow the generation of a high number of recombinant microbial strains in a short amount of time within a single metabolic engineering project. Typically, the strains are tested in small scale cultivation systems such as microtiter plates or shake flasks, and the best performing ones are further characterized at a bioreactor scale. However, due to the development of high throughput (HT) bioreactor platforms, it is now possible to characterize a whole set of genotypic variants at the fluxomic level providing the most complete and systematic description of the metabolic state of a cell [15–17]. A strain’s fluxome is a result derived from the combination of its genome, transcriptome, proteome, and metabolome, and the regulatory interactions between these components. Thus, fluxomics can be used to identify regulatory mechanisms that may be hampering a strain’s performance [18, 19].

**Fig. 1:**
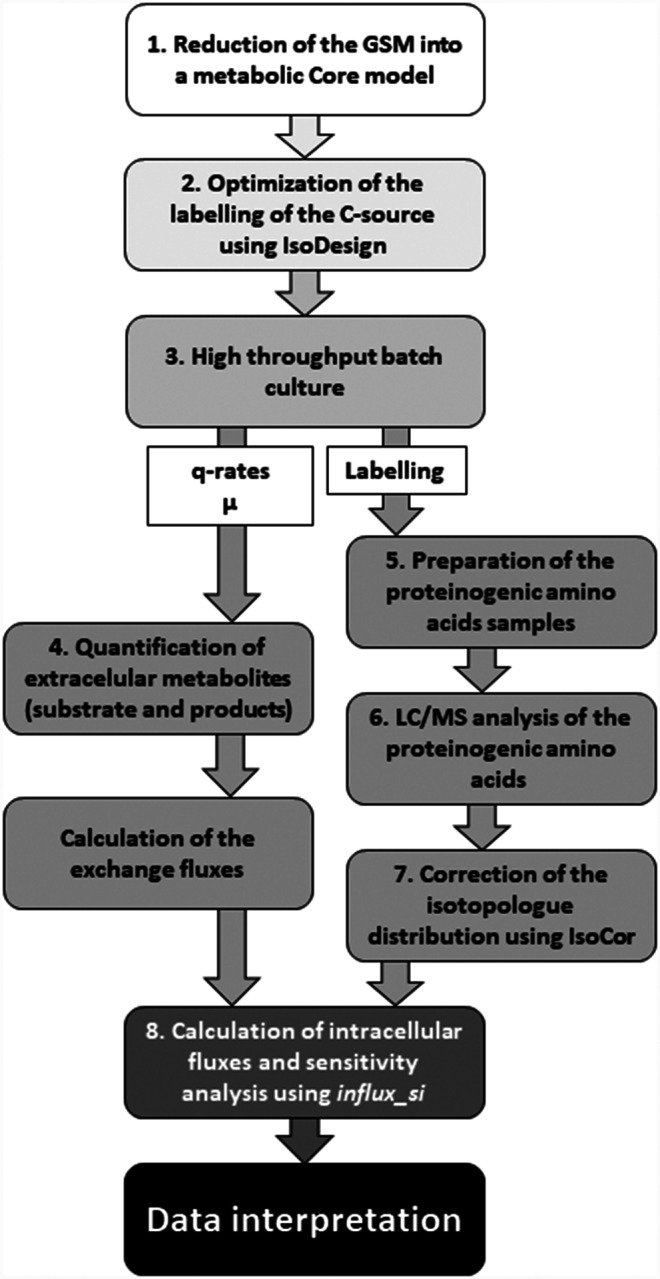
Diagram illustrating the complete workflow for conducting high throughput metabolic flux profiling on a set of *Pichia pastoris* strains. The diagram encompasses all essential steps, starting from model generation and C-source labelling optimization to metabolic flux calculation

^13^C-Metabolic Flux Analysis (^13^C-MFA) is the most widespread technique for the quantification of the fluxes [20]. It consists in using a ^13^C-labelled substrate as tracer to latter infer the metabolic reaction rates. HT analysis of multiple strains has focused on stationary ^13^C-MFA (i.e., cells are collected at a metabolic and isotopic steady state) because the sampling protocol is easier to automatize compared to instationary ^13^C-MFA, as there is a remarkably lower number of samples to process and analyse [16]. The use of ^13^C-MFA for the characterization of *P. pastoris* fluxes has been largely reported using glucose, glycerol, or combinations of thereof with methanol [21–23]. Nonetheless, the exploitation of HT ^13^C-MFA tools and methodologies for the characterisation of *P. pastoris* metabolism remains largely unexplored. In this study, we have applied a workflow that allows the characterisation of the fluxome of multiple strains in parallel. Such workflow integrates all relevant steps from experimental design to data acquisition and processing, and flux calculation (Fig. 1), providing detailed supporting information to facilitate its transferability to other investigations. Notably, the workflow includes the generation of a genome-scale reduced metabolic model applying a compression protocol to a previously described Genome Scale Metabolic (GSM) model of *P. pastoris* [23, 24]. The aim of this study was to understand the impact of different genetic modifications into the fluxome of a set of *P. pastoris* strains that produce 3-HP using glycerol as a sole carbon source. The fluxomics analyses provided meaningful insights of the bottlenecks of this yeast’s metabolism towards 3-HP production. Moreover, the fluxome of the 3-HP producing strains was also characterized at pH 3.5, a relevant condition for industrial production of carboxylic acids.

## Materials and methods

### Strains

The *P. pastoris* strains used in this study are listed in Table 1. These were all derived from the parental strain X-33 (Invitrogen-Thermo Fisher Scientific, MA, USA), which was used as a reference strain for this study. The construction of all strains has been previously described elsewhere [4, 5].

**Table 1:** Strains used in this study. The genotype indicates the overexpressed genes with the corresponding promoter, and the deleted genes

Strain name	Genotype	Reference
X-33		Invitrogen-Thermo Fisher Scientific
PpHP1	pGAP_*mcr*_*Ca*_	[4]
PpHP2	pGAP_*mcr-N*_*Ca*_ pGAP_*mcr-C*_*Ca*_	[4]
PpHP5	pGAP_*mcr-N*_*Ca*_ pGAP_*mcr-C*_*Ca*_ pGAP_*cPOS5*_*Sc*_ (Genbank’s [25] accession number ON52858*)	[4]
PpHP6	pGAP_*mcr-N*_*Ca*_ pGAP_*mcr-C*_*Ca*_ pGAP___*ACC1*_*Y*l_ pGAP_*cPOS5*_*Sc*_	[4]
PpHP8	pGAP_*mcr-N*_*Ca*_ pGAP_*mcr-C*_*Ca*_ pGAP_*mcr-C*_*Ca*_ pGAP___*ACC1*_*Y*l_ pGAP_*cPOS5*_*Sc*_	[5]
PpHP13	pGAP_*mcr-N*_*Ca*_ pGAP_*mcr-C*_*Ca*_ pGAP_*mcr-C*_*Ca*_ pGAP___*ACC1*_*Y*l_ pGAP_*cPOS5*_*Sc*_ pTEF1_*acs*_*Se*_^*L641P*^	[5]
PpHP15	pGAP_*mcr-N*_*Ca*_ pGAP_*mcr-C*_*Ca*_ pGAP_*mcr-C*_*Ca*_ pGAP___*ACC1*_*Y*l_ pGAP_*cPOS5*_*Sc*_ ∆*ArDH*	[5]
PpHP17	pGAP_*mcr-N*_*Ca*_ pGAP_*mcr-C*_*Ca*_ pGAP_*mcr-C*_*Ca*_ pGAP___*ACC1*_*Y*l_ pGAP_*cPOS5*_*Sc*_ pTEF1_*acs*_*Se*_^*L641P*^ ∆*ArDH*	[5]
PpHP18	pGAP_*mcr-N*_*Ca*_ pGAP_*mcr-C*_*Ca*_ pGAP_*mcr-C*_*Ca*_ pGAP___*ACC1*_*Y*l_ pGAP_*cPOS5*_*Sc*_ pTEF1_*acs*_*Se*_^*L641P*^ ∆*ArDH* pPDC1_*PDC1*	[5]

*Available from: https://www.ncbi.nlm.nih.gov/nuccore/ON528582.

### Model generation

The genome scale model *iMT1026v3* [24, 26] was compressed using *CellNetAnalyzer* 2021.1 [27] in Matlab R2020b (Matlab inc., Mathworks, MA, USA). A contextualized core model was generated conserving the reactions, metabolites, and phenotypes that had been previously described for *P. pastoris* grown on glycerol as sole carbon source [23]. Additionally, D-arabitol secretion was conserved, as it has been previously reported that theses strains produce D-arabitol as by-product in both batch and fed-batch cultures [4]. Cytosolic acetate production was also kept during model compression, as cytosolic acetate is used as substrate for 3-HP production. The models and scripts to compress *iMT1026v3* model into a core model can be found in the Supplementary File S1.

The newly generated core model was manually curated considering relevant literature-based knowledge. First, production of isoleucine consumes mitochondrial pyruvate instead of 2-(α-hydroxyethyl)thiamine diphosphate. The production of lysine uses mitochondrial acetyl-CoA and α-ketoglutarate from the cytosol, instead of vice versa. Finally, alanine production is derived from mitochondrial pyruvate, instead of cytosolic pyruvate [28–30]. In addition, as biosynthesis of glutamate, glutamine, aspartate, and asparagine may take place in both the cytoplasm and the mitochondria, both pathways were included. Finally, the non-oxidative branch of the pentose phosphate pathway was described using the half-reactions model, which considers the kinetic mechanism of the transketolase and transaldolase reactions [31]. The 3-HP production pathway from cytosolic acetate was also included.

All the necessary files to generate the new core model (*PpaCore_3HP.mat*) from the GSM model can be found in the Supplementary File S2. The final core stoichiometric model contains 151 species and 145 reactions.

For ^13^C-flux calculations, some of the amino acid biosynthetic reactions were manually lumped to reduce the number of overall reactions and metabolites. Some of the intermediary metabolites of the lumped reactions were present in the biomass production reactions. The stoichiometry of the biomass formation equation was corrected to remove these metabolites. These modifications can be found in the Supplementary File S3. This model was converted into FTBL (Flux TaBuLar) format [20, 32]. The reactions producing 3-HP from cytosolic acetate (the closest intermediary metabolite to malonyl-CoA in the core model) were introduced, and carbon atom transitions were added for all reactions to simulate label propagation. The final core isotopic model for the *P. pastoris* 3-HP producing strains, which contains 113 species and 123 reactions, can be found in the Supplementary File S4* (PpaCore_3HP.ftbl*). The FTBL model can also be obtained from BIOMODELS [33], accession number: MODEL2210090004.

### *In silico* design of ^13^C-labelling experiments

The context-specific core model of *P. pastoris* metabolism including the 3-HP formation reactions (*PpaCore_3HP.ftbl*) was used to calculate the optimal isotopic composition of the substrate using *IsoDesign* v1.2.1 [34]. *IsoDesign* uses *influx_si* [35] to calculate the precision of the fluxes for each (mixture of) label input to be tested.

To determine the optimal label input, the ^13^C-glycerol combinations yielding the highest number of fluxes with a SD < 1 was calculated. The commercially available ^13^C-labelled glycerol variants include 1-^13^C-glycerol, 2-^13^C-glycerol, 1,3-^13^C-glycerol, and U-^13^C-glycerol. The substrate combinations were ranked based on the sum of SDs of the reactions with a SD < 1. Afterwards, the substrates providing the highest precision of the fluxes of the upper glycolysis (UG) and the pentose phosphate pathway (PPP) were ranked. The IsoDesign results indicated that the best substrate combination consisted of 20% 1-^13^C-glycerol and 80% 2-^13^C-glycerol (Supplementary File S5).

### Media, cultivation conditions, and automated sampling

Seed cultures for the mini-bioreactor cultivations were prepared as follows: 50 mL falcon tubes containing 5 mL YPG (1% w/v yeast extract, 2% w/v peptone, and 1% v/v glycerol) medium were inoculated from cryostock and incubated overnight at 30^o^C and 200 rpm. The overnight cultures were transferred to a 250 mL shake flask containing 25 mL YPG at a starting OD_600_ of 1 and subsequently grown for 8 h at 30^o^C and 200 rpm. The cultures were harvested at the exponential phase and used to inoculate the mini-bioreactor cultures.

A HT fluxomics platform consisting of 48 mini bioreactors was used for the cultivation and the sampling [36]. Briefly, the 50-mL bioreactors of this platform are equipped with pH and dissolved oxygen sensors for process monitoring and control. The bioreactors are agitated using a magnetic stirrer bar and they are designed to be aerated through the headspace of each reactor to avoid the accumulation of CO_2_.

The batch medium contained 1.8 g L^− 1^ citric acid, 0.02 g L^− 1^ CaCl_2_ · 2 H_2_O, 12.6 g L^− 1^ (NH_4_)_2_HPO_4_, 0.5 g L^− 1^ MgSO_4_ · 7 H_2_O, 0.9 g L^− 1^ KCl, 0.4 mg L^− 1^ biotin, and 4.6 ml L^− 1^ of PTM1 trace salts [37]. The pH of the medium was adjusted to 5 or 3.5 using HCl. The batch medium was supplemented with 0.5 g L^− 1^ glycerol labelled at position 1 and 2 g L^− 1^ glycerol labelled at position 2 (20% 1-^13^C-glycerol and 80% 2-^13^C-glycerol). Labelled substrates were obtained from Innovachem SAS (France). All the components of the medium except for the trace salts, the biotin, and the labelled glycerol were mixed and autoclaved. The other components were filter-sterilized using a syringe filter with 0.2 μm pore size and introduced into the batch medium under sterile conditions. Each bioreactor was filled with 15 mL of medium.

All the strains listed in Table 1 were tested in triplicate in bioreactor cultures at pH 5. The cultures were inoculated at a starting OD_600_ of 0.025. The strains X-33, PpHP1, PpHP6, PpHP8, PpHP15, and PpHP18 were tested in triplicate at pH 3.5. The cultures were inoculated at a starting OD_600_ of 0.05. The temperature was set to 28^o^C. The stirring rate was set to 2200 rpm. The air flow into the sterile gas cover, which ensures identical gas distribution into the head spaces of all the 48 bioreactors, was controlled to 104 mL min^− 1^ per bioreactor by a mass flow controller (Aalborg Instruments, NY, USA).

Automated sampling was programmed to measure the OD_600_ off-line every two hours for the first 16 h of cultivation. After 16 h of cultivation, sampling of the OD_600_ was performed every hour (during the exponential phase of the cultures). From the 16 to the 24 h of cultivation, 250 µL samples were withdrawn hourly from each culture for supernatant analysis. The samples were placed on 96-well plates with a 0.45 μm filter bottom and vacuum filtered immediately. A final supernatant sample was withdrawn after 27 h cultivation. After 27 h cultivation, OD_600_ samples were withdrawn every 2.5 h for 10 h. Sampling and OD_600_ data from the mini-bioreactor cultures can be found on Supplementary File S6. When each culture reached an OD_600_ above 1, a 500 µL sample was withdrawn from the culture. The samples were quenched on 3.5 mL quenching solution (40% v/v acetonitrile, 40% v/v methanol, 20% water and 0.1 M formic acid) at -20^o^C. Samples were stored at -20^o^C between 1 and 3 h until further processing.

### Analyses

#### Exometabolites analysis with NMR

Glycerol, 3-HP, and D-arabitol were quantified from the filtered supernatant samples using Nuclear Magnetic Resonance (NMR). 180 µL of sample and 20 µL of 10 mM TSP (3-(trimethylsilyl)-[2,2,3,3-^2^H_4_]-propionic acid sodium salt) were mixed. TSP was used as internal standard and quantified using a commercial succinate solution at 1 g L^− 1^ (43,057, Sigma Aldrich, MO, USA). 1D-^1^ H analyses were carried out on a Bruker Advance III 800 MHz spectrometer (Bruker BioSpin, Germany) equipped with a 5 mm CQPI cryoprobe. Samples were analysed at 280 K using a 30^o^ pulse sequence with water suppression (zgpr30), with a relaxation delay of 7 s. TopSpin v3.6.4 (Bruker BioSpin, Germany) was used for the analysis of the NMR spectra. Commercial succinate at 1 g L^− 1^ (43,057, Sigma Aldrich, MO, USA) was used for the re-quantification of TSP. All data was corrected based on the quantification of succinate. The peak integration data from the NMR analyses can be found in Supplementary File S7.

#### Sample processing and analysis of the ^13^C-proteinogenic amino acids using LC/MS

Quenched samples were vortexed for 30 s and centrifuged at 5,000 g for 5 min in a swing-rotor centrifuge. Pellets were dried using a Rotavapor (Büchi, Switzerland). The biomass was treated with 150 µL 6 N HCl at 110 °C for 16 h. The acid was evaporated in the rotavapor. Each sample was washed using 100 µL milliQ water and subsequently dried in the rotavapor. This step was repeated twice to eliminate all the acid traces. Finally, the pellets were resuspended on 200 µL milliQ water. The samples were centrifuged at 10,000 g for 5 min to eliminate the biomass debris. 40 µL of supernatant (containing the amino acids) were diluted onto 460 µL milliQ water.

The diluted amino acids were analyzed using a previously described HPLC-MS method [38]. The UHPLC Vanquish (Thermo Fisher Scientific, MA, USA) was coupled to a mass spectrometry (MS) detector Orbitrap Q-Exactive plus with a heated electrospray ionization source (Thermo Fisher Scientific). HPLC-MS was used with a precolumn Discovery HS F5 Supelguard Cartridge of 20 × 2.1 mm with particle size 5 μm (Supelco Bellefonte, PA, USA) and a column Discovery HS F5 HPLC column of 150 × 2.1 mm with particle size 5 μm (Supelco Bellefonte). Solvent A consisted of 0.1% v/v formic acid in ultrapure water and solvent B of 0.1% v/v formic acid in acetonitrile. The flow rate of the eluent was set to 0.25 mL min^− 1^, and the temperature of the sampler and the column were set to 4 and 30 °C, respectively. Solvent B set points were varied as follows: 0 min: 2%; 2 min: 2%; 10 min: 5%; 16 min: 35%; 20 min: 100%; 24 min: 100%. Finally, the set points of the initial conditions (2% solvent B) were set for 6 min before the injection of the next sample. The injection volume was 5 µL.

The MS detector was set to detect the positive ions on FTMS mode. The resolution was set to 70,000 (at m/z = 400), the capillary temperature to 320 °C, and the source heater temperature to 300 °C. Sheath gas and auxiliary gas flow rates were set to 40 and 10 arbitrary units, respectively. The S-lens RF level was set to 40% and the source voltage to 5 kV. The MS was set to measure the exact mass of all carbon isotopologues of all the amino acids (Supplementary File S8).

The isotopologue distribution of the amino acids was corrected considering the isotopologue labelling of the inoculum and the natural abundance of all isotopes using IsoCor v2.2.0 (https://github.com/MetaSys-LISBP/IsoCor) [39]. Raw and processed data can be found in Supplementary file S9.

### Bioprocess parameters

The OD_600_ and the NMR supernatant analyses results were used to calculate the growth rate (µ) and the specific production or consumption rates (q-rates) for glycerol, 3-HP, and D-arabitol. To this end, PhysioFit v1.0.2 (https://github.com/MetaSys-LISBP/PhysioFit) was used [40]. The absorbance at OD_600_ was correlated to the biomass concentration using a constant conversion factor for all the strains, the rationale being that all the strains used in this study shared the same conversion factor in the culture conditions used. This assumption is supported by experimental data from our previous studies [4, 5], where the conversion factor of the biomass absorbance (OD_600_) to the cell dry weight (CDW) was statistically identical for strains PpHP6, PpHP8, and PpHP18 in batch cultures using the same growth medium. The conversion factor value was derived from calculating the µ (which is independent from the conversion factor) of the triplicate cultures of the reference strain (X-33) at pH 5. Afterwards, the genome scale model *iMT1026v3* was used to calculate the uptake rate of glycerol at such growth rate (0.21 h^− 1^) considering a non-growth associated ATP consumption of 2.51 mmol g_CDW_^−1^ h^− 1^ [24], which resulted 3.4 mmol_Glyc_ g_CDW_^−1^ h^− 1^. This calculation was done in Matlab using the CobraToolbox v2.26.0 [41]. Finally, the conversion factor that correlates the experimental data and the computational results was calculated to be 0.563 g_CDW_/OD_600_. The biomass yield for strain X-33 using this calculated conversion factor resulted in 0.73 g_CDW_ g_Glyc_^−1^, which falls within the reported experimental values [24].

The biomass concentration data can be found together with the OD_600_ data in Supplementary File S6.

The bioprocess parameters results calculated using PhysioFit can be found in the Supplementary File S10.

### ^13^C-Flux calculation

Flux calculations were performed from metabolic and isotopic steady-state data using *influx_si* v5.3.0 (https://influx-si.readthedocs.io, [35]) and the previously obtained FTBL format model. The bioprocess parameters and the corrected data of the isotopologue distribution of the amino acids were used as input data. All fluxes could be determined accurately from the extracellular fluxes and labelling data except for fluxes through the MDHc, MDHm, Tr_AKGMALtm and Tr_AKGOAAtm reactions.

All fluxes were normalized to the rate of the substrate uptake rate. A sensitivity analysis was performed using the Monte-Carlo method with 100 independent simulations. Statistical analysis (chi-square test) was used to determine sufficient goodness of the fit (based on a 95% confidence level) for each experiment. The average fluxes and associated standard deviations of the mean were calculated from the biological replicates with sufficient goodness of the fit.

Throughout this study, the reference guidelines for the calculation of fluxes derived from ^13^C-data have been followed [42]. Therefore, all the relevant data is adequately shared to the community for reproducibility and further analyses.

Flux distributions were visualized using Omix v2.0.7 (Omix Gmbh, Germany) [43]. The figures show the average absolute or relative fluxes and the standard deviation of the triplicates.

### Flux balance analysis

Flux Balance Analysis (FBA) [44] was performed using the CobraToolbox v2.26.0 in Matlab R2020b and the previously described *PpaCore_3HP.mat* model. The experimental confidence intervals obtained from the Monte Carlo analyses were used as upper and lower bounds for each flux. Afterwards, FBA was performed using the maximization of the flux of the ATP of maintenance reaction (ATPM) as objective function. This is the objective function resulting in the best description of the intracellular fluxes of a cell culture growing on a batch culture with excess of substrate [45]. FBA results were also used to calculate the ATP and NADPH balance by summing fluxes through all producing and consuming reactions for each strain. The Matlab script, the input file with the Monte Carlo results, and the FBA results file can be found in the Supplementary File S11.

## Results and discussion

### HT cultivation bioprocess parameters

**Fig. 2:**
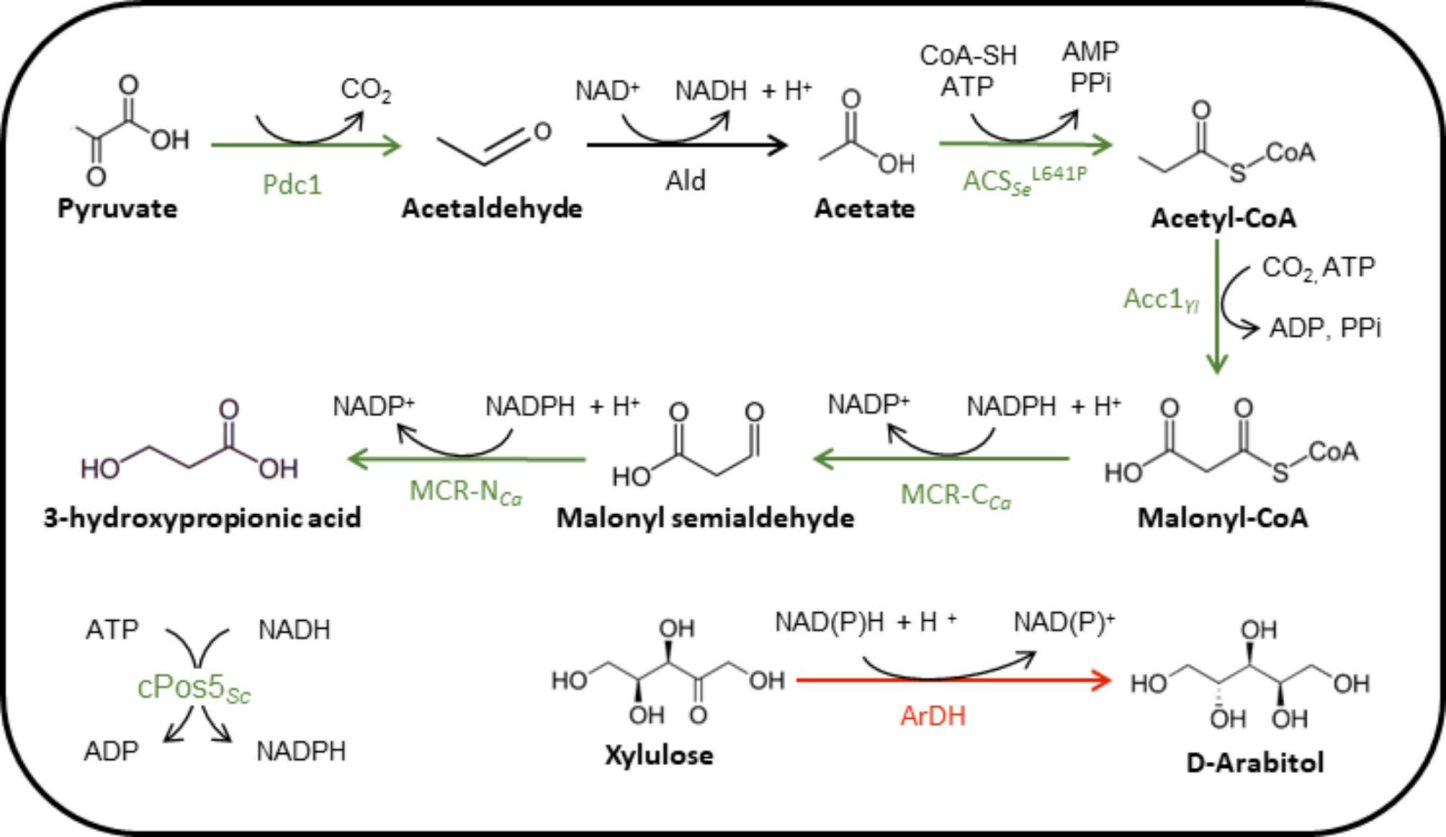
Metabolic pathway from pyruvate to 3-HP through cytosolic acetyl-CoA and malonyl-CoA in *P. pastoris*. The reactions catalysed by all the enzymes linked to overexpressed or deleted genes are displayed. Enzyme abbreviations: Pdc1: Pyruvate decarboxylase 1; Ald: Endogenous cytosolic aldehyde dehydrogenase; ACS_Se_^L641P^: Acetyl-CoA synthase from *Salmonella enterica* harbouring the point mutation L641P to avoid post-translational inhibition of the enzyme by acetylation; Acc1_*Yl*_: Acetyl-CoA carboxylase from *Y. lipolytica*; MCR-C_*Ca*_: C-terminal domain of the malonyl-CoA reductase from *C. aurantiacus*; MCR-N_*Ca*_: N-terminal domain of the malonyl-CoA reductase from *C. aurantiacus*; cPos5_*Sc*_: NADH kinase from *S. cerevisiae* located on the cytosol; ArDH: Arabitol dehydrogenase. Green and red arrows and enzyme abbreviations indicate whether the corresponding genes were overexpressed or deleted, respectively

The series of previously available 3-HP-producing *P. pastoris* strains[4, 5] were grown in glycerol batch cultures at pH 5 using a HT ^13^C-fluxomics platform [36]. The stoichiometry of the engineered reactions and corresponding enzymes in the 3-HP-producing *P. pastoris* strains are depicted in Fig. 2. Strain engineering strategies were aimed at increasing the delivery of the substrates of the malonyl-CoA to 3-HP pathway (i.e., cytosolic acetyl-CoA, malonyl-CoA, and NADPH), and the reduction of the production of the main by-product (D-arabitol).

**Fig. 3:**
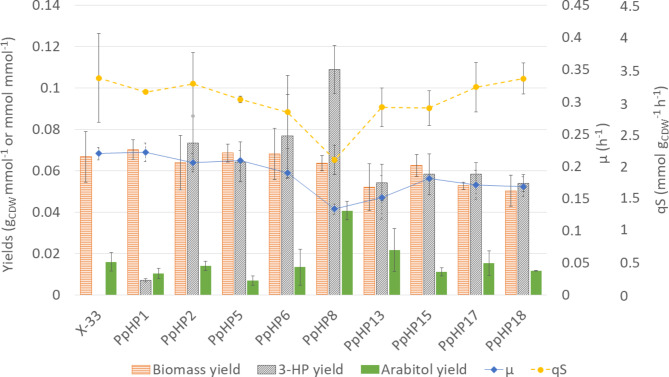
Bioprocess parameters of the parental *P. pastoris* strain and nine 3-HP-producing strains cultivated in glycerol batch mini bioreactor cultures at pH 5. Orange bars show the biomass yield, grey bars show the 3-HP yield, green bars show the D-arabitol yield, blue diamond depict the µ_max_, and yellow circles show the substrate uptake rate (qS). Standard deviation of the replicates is depicted

Measurement of the extracellular metabolites and the biomass concentration allowed calculating the bioprocess parameters (specific substrate consumption and (by)products production rates (q-rates) and µ_max_) for each strain (Fig. 3), which is a prerequisite for metabolic flux calculations. Results were consistent with previously reported cultivation data [4, 5]. In a nutshell, comparison between strains PpHP1 and PpHP2 reveals that dissection of MCR from *C. aurantiacus* into each of its two subunits resulted in increased specific malonyl-CoA reductase activity [4, 46], hence leading to a 10-fold increase in the 3-HP yield. The overexpression of the genes encoding for an acetyl-CoA carboxylase from *Yarrowia lipolytica* (*ACC1*_*Yl*_) and a cytosolic version of the mitochondrial NADH kinase from *Saccharomyces cerevisiae* (*cPOS5*) in strain PpHP6 led to a further increase in 3-HP yield by enhancing the conversion of acetyl-CoA into malonyl-CoA (3-HP’s metabolic precursor) and NADPH supply, respectively. In addition, the expression of a second copy of the gene encoding the C-terminal domain of MCR yielded the highest 3-HP-producing strain (PpHP8), which was also the slowest growing strain and the strain producing the largest amount of D-arabitol in batch cultures. As previously reported, the overexpression of the genes encoding for the cytosolic acetyl-CoA synthesis pathway (i.e., *acs*_*Se*_^*L641P*^ and *PDC1*, Fig. 2) in the strain PpHP8 (yielding strain PpHP18), aimed to increase the conversion of pyruvate to cytosolic acetyl-CoA, restored the growth rate, but the 3-HP yield dropped drastically [5]. These results pointed at a limitation of resources in PpHP8 when growing at maximal growth rate [5], thereby resulting in an opposite trend between biomass and product yields.

Results in Fig. 3 also show that not only the growth rate of strain PpHP8 was remarkably lower than the growth rate of the reference strain (0.13 h^− 1^ and 0.22 h^− 1^, respectively), but also the substrate uptake rate (2.10 and 3.37 mmol gCDW^− 1^ h^− 1^). In fact, the glycerol specific uptake rate (q_S_) of PpHP8 was significantly lower than the mean q_S_ value observed for the rest of strains (based on a one-way ANOVA test, p = 0.02). A high activity of the malonyl-CoA to 3-HP pathway probably led to a reduced availability of acetyl-CoA for this strain. Acetyl-CoA plays a central role on the biosynthesis of precursors, and it plays a key role in physiological regulation processes, such as the acylation of histones [47]. Growth defects have also been reported in *S. cerevisiae* strains harbouring a high acetyl-CoA carboxylase activity, where depletion of acetyl-CoA was described as the most likely cause of hampered growth [48]. Moreover, the observed increase in the growth rate and the uptake rate when *acs*_*Se*_^*L641P*^ was overexpressed support this hypothesis.

Strikingly, the biomass yield for PpHP8-derived strains overexpressing *acs*_*Se*_^*L641P*^ (PpHP13, ppHP17), and *PDC1* plus *acs*_*Se*_^*L641P*^ (PpHP18) were lower than the biomass yield of strain PpHP8.

Notably, all strains showed production of D-arabitol under the tested conditions. This by-product is typically produced due to an imbalance on the NADPH regeneration [4, 5], resulting in reduced biomass and product yields. Strikingly, deletion of the main D-arabitol dehydrogenase encoding gene (*ArDH*) in strains PpHP15 and PpHP17 resulted in increased growth rates but reduced 3-HP production, compared to their corresponding parental strains (PpHP8 and PpHP13, respectively).

### **Fluxome of*P. pastoris*3-HP-producing strains at pH 5**

The bioprocess parameters of the series of strains obtained from batch cultivations and the amino acids isotopologue distributions derived from the corresponding labelling experiments were used to calculate the intracellular fluxes. The fluxome of each strain can be found in the Supplementary File S12 (see also Supplementary File S13 for a summary of the results).

The metabolic flux profile of the reference strain (X-33) obtained using the HT robotic platform is comparable to the previously reported results for the same strain growing in glycerol chemostats on a similar medium [23]. Noticeably, the fluxes of the upper glycolysis (UG) and pentose phosphate pathway (PPP) in our batch experiments (i.e., at µ_max_) were higher than the ones observed in chemostat cultures at lower growth rates (0.10 and 0.16 h^− 1^), coherent with the positive correlation between growth rate and the UG and PPP fluxes previously observed in glycerol chemostats (Additional Figure S1). Similarly, fluxes of the lower glycolysis (LG) and the tricarboxylic acid (TCA) cycle reactions were lower than the ones observed in chemostat cultures at lower growth rates, also coherent with the reported inverse correlation between growth rate and LG and TCA cycle fluxes. Lower LG and TCA cycle fluxes are consistent with increased UG and PPP fluxes, as already reported [23].

**Fig. 4:**
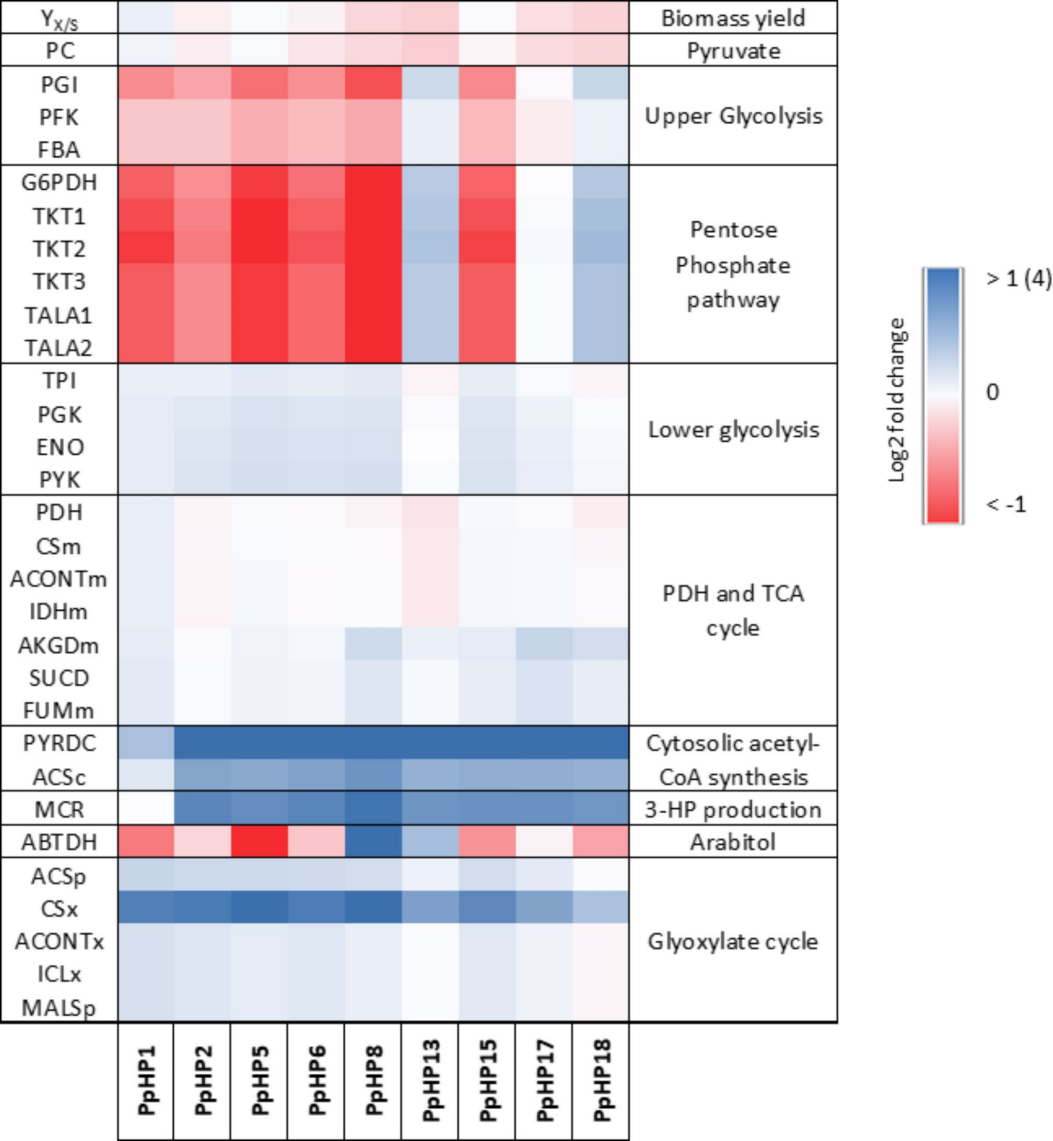
Reaction flux Log_2_ fold-change between each one of the ten 3-HP-producing recombinant strains compared to the reference strain (X-33). All colours are referred to the − 1 to + 1 colour scale, except the flux of the ‘MCR’ reaction, where the upper boundary of the colour scale is set to + 4. Moreover, in the case of ‘MCR’, the Log_2_ fold-change of the fluxes is referred to the flux of strain PpHP1. Y_X/S_ is the biomass to substrate yield. The reaction stoichiometry corresponding to each reaction abbreviation can be found in the Supplementary File S3

To facilitate comparison of metabolic flux distributions amongst strains, a heat map illustrating the fold-change between the relative fluxes (i.e., normalised to the specific glycerol uptake rate) of each recombinant strain compared to the relative fluxes of the reference strain cultivated at pH 5 is shown in Fig. 4. The most drastic changes were observed in the relative fluxes through the UG and PPP reactions. First, when the MCR activity was increased by expressing separately the two MCR domains (i.e., PpHP2 compared to PpHP1), the fluxes of the UG and PPP increased noticeably (10∼25%). Such trend can be explained by increased NADPH requirements, as NADPH is used as the electron donor for the two consecutive reactions catalysed by MCR. When the gene encoding the heterologous cytosolic NADH kinase (*cPOS5*_*Sc*_) was overexpressed (PpHP5), the observed fluxes of the UG and PPP reactions were 15∼30% lower compared to PpHP2. It is well known that the NADPH/NADP^+^ ratio controls the fluxes towards the oxidative branch of the PPP [49]. In addition, overexpression of *cPOS5* in *P. pastoris* provides the cell with an additional source of NADPH, leading to a higher NADPH/NADP^+^ ratio [50]. Therefore, decrease of UG and PPP fluxes in PpHP5 compared to PpHP2 was consistent with an increased NADPH/NADP^+^ ratio.

No major changes were observed when *ACC1*_*Yl*_ was overexpressed (i.e., in strain PpHP6, compared to strain PpHP5). For the strain PpHP8, which harboured an additional copy of the gene encoding for MCR-C_*Ca*_, the highest fluxes towards 3-HP production were observed, while the UG and PPP fluxes were the lowest among all strains. Such observation agrees with the results for the strain PpHP5, as the increase in the NADPH requirements due to production of 3-HP followed independent trends with the fluxes of the oxidative branch of the PPP.

Heterologous expression of *acs*_*Se*_^*L641P*^ in strain PpHP8 (i.e., generating strain PpHP13) led to a drastic switch in the strain’s fluxome. The relative fluxes through the UG and PPP increased remarkably in PpHP13, while showing a lower relative flux towards 3-HP production compared to PpHP8 or PpHP6 strains. Moreover, the biomass yield of PpHP13 was lower. Therefore, considering the NADPH requirements for biomass and 3-HP production, increased production of NADPH through the PPP seems unfounded. Deletion of the gene encoding for the NADPH-dependent arabitol dehydrogenase enzyme (*ArDH*) in strains PpHP8 and PpHP13 (i.e., obtaining strains PpHP15 and PpHP17, respectively) led to minor changes in the strains’ fluxomes under the tested growth conditions. Finally, overexpression of *PDC1* in PpHP17 (resulting in PpHP18) led to the highest relative flux through the UG and PPP. However, neither the biomass yield nor the 3-HP yield were affected (Fig. 3).

Changes in the fluxes through LG and the TCA cycle reactions followed the opposite trend to the UG and the PPP. Small differences in the fluxes through the glyoxylate cycle were also observed. However, as the absolute values of these fluxes were low (below 0.025 mmol mmol^− 1^ h^− 1^), absolute changes of these fluxes did not have an impact on the strain’s biomass and product yields.

**Fig. 5:**
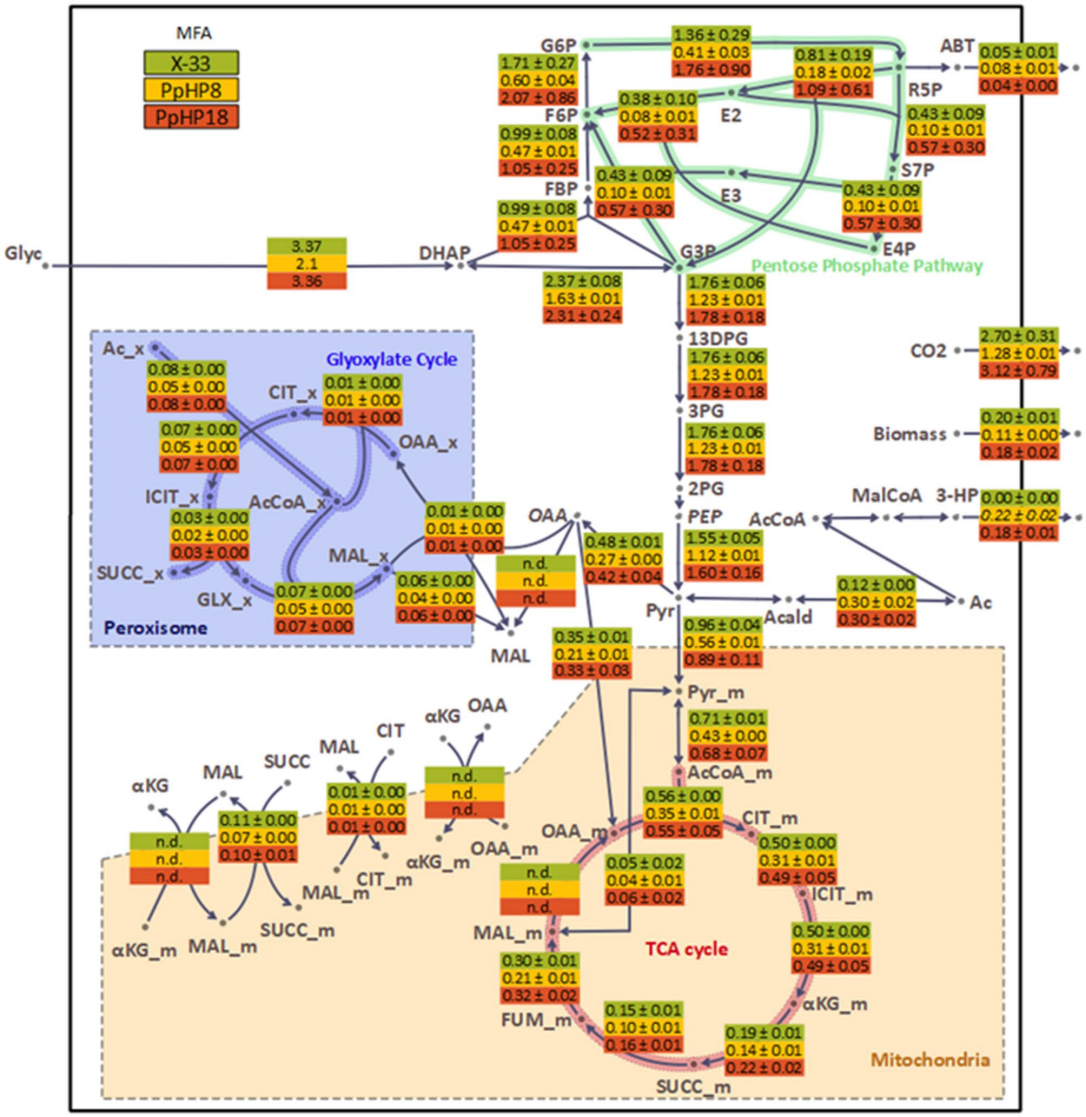
Flux map for the reference (X-33), PpHP8, and PpHP18 strains growing in glycerol batch cultures at pH 5. The average and the standard deviation of the absolute fluxes for each triplicate experiment are displayed. Fluxes are given in mmol gCDW^− 1^ h^− 1^. Abbreviations: G6P: Glucose-6-phosphate; F6P: Fructose-6-phosphate; FBP: Fructose bisphosphate; Glyc: Glycerol; DHAP: Dihydroxyacetone phosphate; G3P: Glyceraldehyde-3-phosphate; R5P: Ribose-5-phosphate; S7P: Sedoheptulose-7-phosphate; E4P: Erythrose-4-phosphate; ABT: D-arabitol; E2: glycolaldehyde moiety of the non-oxidative PPP reactions; E3: dihydroxyacetone moiety of the non-oxidative PPP reactions; 13DPG: 1,3-Bisphosphoglycerate; 3PG: 3-phosphoglycerate; 2PG: 2-phosphoglycerate; PEP: Phosphoenolpyruvate; Pyr: Pyruvate; Acald: Acetaldehyde: Ac: Acetate; AcCoA: Acetyl-CoA; MalCoA: Malonyl-CoA; 3-HP: 3-Hydroxypropionic acid: CIT: Citrate; ICIT: Isocitrate; αKG: α-ketoglutarate; SUCC: Succinate; FUM: Fumarate; MAL: Malate; OAA: Oxaloacetate; GLX: Glyoxylate

Further comparison of the absolute flux distributions (i.e., non-normalised to the glycerol specific uptake rate) in the reference and the 3-HP-producing strains PpHP8 and PpHP18 (Fig. 5) provided additional insights.

First, overexpression of the 3-HP production pathway led to a higher pyruvate decarboxylase flux at the pyruvate node in PpHP8 compared to the reference strain (0.30 mmol g_CDW_^−1^ h^− 1^ and 0.12 mmol g_CDW_^−1^ h^− 1^, respectively). The overexpression of ACS_*Se*_^L641P^ and Pdc1 in strain PpHP8 did not result in a higher flux towards cytosolic acetyl-CoA in strain PpHP18, as the absolute flux values for the two strains were identical (0.30 mmol g_CDW_^−1^ h^− 1^). Compared to the reference strain, overexpression of the cytosolic acetyl-CoA production pathway in strain PpHP18 did not increase the LG fluxes. All these findings agree with previous results in *S. cerevisiae*, where the overexpression of *PDC1* led to a higher flux towards this pathway without increasing the glycolytic flux [51]. It is well described that the glycolytic flux is tightly controlled in yeast *S. cerevisiae*, and the glycolytic flux cannot be increased by overexpressing individual enzymes [52]. In the case of *P. pastoris*, increased glycolytic fluxes have only been described under low oxygen availability [53] or when a transcription factor controlling the expression of all the glycolytic genes was overexpressed [54]. Therefore, as pyruvate is pulled into the production of 3-HP, but the glycolytic flux and the uptake of glycerol do not increase, the overall ATP yield of the 3-HP-producing strains decreases.

Strain PpHP18 has remarkably higher UG and PPP fluxes than strain PpHP8 (Fig. 5). The UG and PPP have a low carbon and energy yield. Therefore, while *PDC1* is being overexpressed, the energy requirements in strain PpHP18 sinked the pyruvate into the TCA cycle for ATP generation, hampering the flux toward cytosolic acetyl-CoA and, ultimately, reducing the 3-HP yield. On the contrary, as PpHP8 grew at a lower rate, the energy requirements of the strain were reduced, leaving more substrate available to produce 3-HP.

Overall, comparison of absolute flux distributions suggests that to further increase the 3-HP yield in strain PpHP8, the glycolytic fluxes would need to be significantly increased. Moreover, results also point to high ATP requirements in strain PpHP18 are the cause of the differences between these two strains.

### ATP and NADPH producing/consuming fluxes of the 3-HP-producing *P. pastoris* strains

**Fig. 6:**
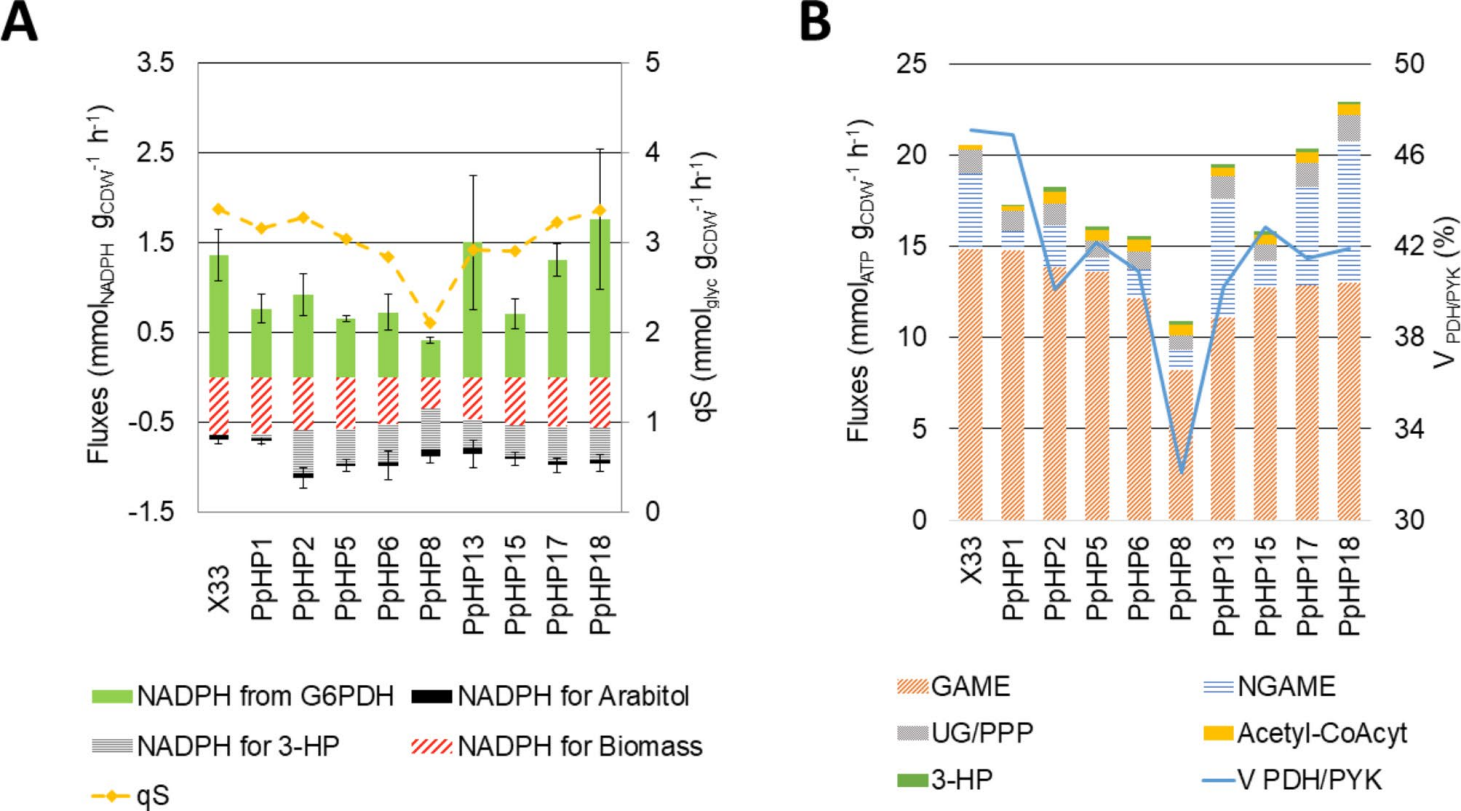
** A.** Production and consumption rates of NADPH (estimated from the ^13^C-MFA results) and specific glycerol uptake rates for each strain. **B.** FBA results. The y-axis on the left side shows the sum of the fluxes for the main ATP-consuming reactions. The y-axis on the right side shows the percentage of pyruvate entering the mitochondria and the TCA cycle. To do so, the ratio of the fluxes of PYK (the main cytosolic pyruvate producing reaction) and PDH was calculated. Abbreviations: G6PDH, glucose-6-phosphate dehydrogenase; GAME, growth associated maintenance energy; NGAME, non-growth associated maintenance energy; UG/PPP: upper glycolysis and pentose phosphate pathway reactions; Acetyl-CoAcyt: Cytosolic acetyl-CoA production pathway; V_PDH/PYK_: Ratio between the fluxes of the pyruvate dehydrogenase (PDH) and the pyruvate kinase (PYK) reactions

The results from the previous section show that the fluxes through the PPP decreased when a heterologous cytosolic NADH kinase was expressed. Consistently, NADPH production rates calculated from the ^13^C-flux data (Fig. 6A) indicate that the NADPH produced by the glucose-6-phosphate dehydrogenase (G6PDH) reaction was lower than the actual NADPH requirements in some of the strains overexpressing the *cPOS5* gene (i.e., strains PpHP5, PpHP6, andPpHP8), supporting that the NADH kinase reaction contributed to cover the cell’s NADPH requirements.

Strains PpHP13, PpHP17, and PpHP18, which overexpress the cytosolic acetyl-CoA production pathway (i.e., Pdc1 and ACS_Se_^L641P^), also produced more NADPH through the oxidative branch of the PPP than the actual cell requirements. The substantial increase in the fluxes through UG and PPP for such strains coincided with an increase in the specific glycerol uptake rate and the growth rate (Fig. 6A, and Fig. 3), but the biomass yield decreased (Fig. 3). Despite the large standard deviation of the fluxes through the UG and PPP fluxes in strains PpHP13, PpHP17, and PpHP18, it can be concluded that these strains did not benefit from the heterologous expression of the cPos5, while PpHP5, PpHP6, and PpHP8 do. NADPH production by NADH kinase is more efficient in terms of both carbon and ATP conservation than the use of the UG and PPP, which would explain the reduction in the biomass and 3-HP yield in the strains PpHP13, PpHP17, and PpHP18.

To corroborate the consistency of the observed metabolic fluxes, FBA was used to verify the redox and energy conservation of the ^13^C-MFA results. The maximization of the flux through the ATP sink reaction (ATPM) was used as an objective function. This is the function best describing the intracellular fluxes of a cell culture growing on a batch culture with an excess of substrate [45]. Consequently, the resulting flux values are based on in vivo data, and at the same time, they fulfil the biological function of optimizing the biomass yield. FBA results confirmed that both redox and energy balances could be conserved at the given experimental fluxes. The FBA results were also used to calculate the ATP balance (Fig. 6).

A higher ATP of maintenance for strains PpHP13, PpHP17, and PpHP18 was observed, as depicted in Fig. 6B, being PpHP18 the strain with the highest ATP requirements among all strains. Conversely, the calculated ATP requirement for strain PpHP8 was the lowest, particularly due to the lowest growth associated maintenance energy (GAME) requirements, as it is the slowest growing strain.

Figure 6B shows there is a direct correlation between the overall ATP requirements of the strain and the fraction of pyruvate that was directed into the TCA cycle for ATP production. While 47.1% of the pyruvate was channelled into the mitochondria in the reference strain, 32.1% was directed through the same pathway in strain PpHP8. The mitochondrial transport of pyruvate raised to 40.2% and 41.9% when the cytosolic acetyl-CoA pathway was expressed (strains PpHP13 and PpHP18, respectively) to compensate for the higher ATP requirements.

Altogether, these results show the correlation between acetyl-CoA availability, growth rate, ATP requirements, and flux distribution at the pyruvate node, and their impact on the 3-HP production yield. Acetyl-CoA depletion in strain PpHP8 hampered growth rate. When the cytosolic acetyl-CoA biosynthetic pathway was overexpressed (strains PpHP13, PpHP17, and PpHP18), growth rate increased. Increase in the growth rate increased ATP requirements, which increased channelling of pyruvate into the TCA cycle, hampering the 3-HP product yield.

### Fluxome of *P. pastoris* strains at pH 3.5

It has been reported that a pH of 3.5 (i.e., 1 unit below the pK_a_ of 3-HP) was optimal for the further downstream processing of 3-HP by solvent extraction [12]. Therefore, the reference strain (X-33) as well as some 3-HP-producing strains (i.e., PpHP1, PpHP6, PpHP8, PpHP15, and PpHP18) were further tested at pH 3.5.

**Fig. 7:**
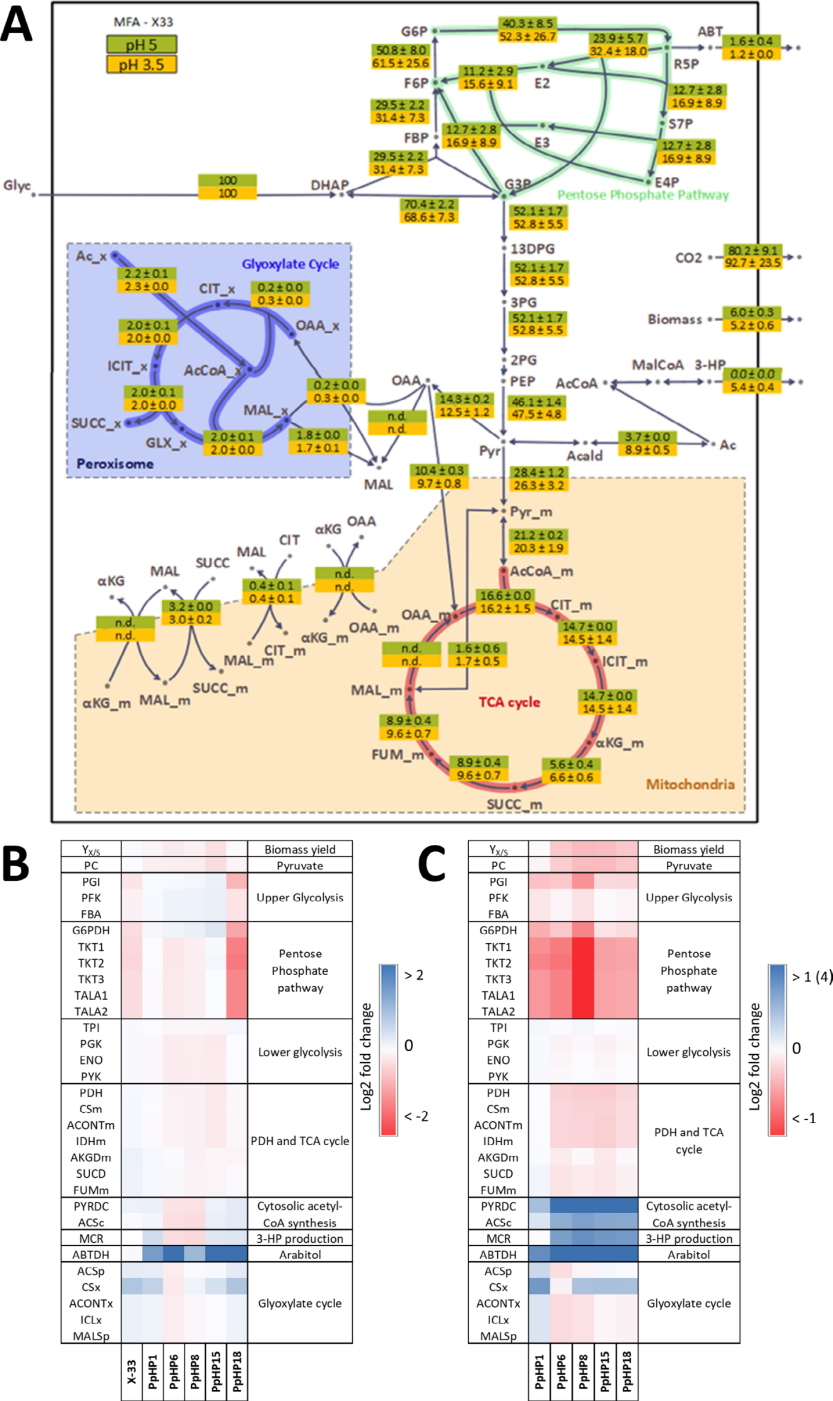
** A.** Flux map with the relative fluxes for the parental strain grown at pH 5 and pH 3.5. Metabolite abbreviations can be found in the caption of Fig. 5. **B.** Log_2_ fold-change of the relative fluxes at a culture pH of 3.5 of the strains X-33, PpHP1, PpHP6, PpHP8, PpHP15, and PpHP18 compared to the same strains grown at pH 5. The colour scale describes the Log_2_ fold-change at a -2 to 2 range. **C.** Log_2_ fold-change of the relative fluxes at a culture pH of 3.5 of the strains PpHP1, PpHP6, PpHP8, PpHP15, and PpHP18 compared to the parental strain. The colour scale describes the Log_2_ fold-change at a -1 to 1 range. ‘MCR’ fluxes fold-change are compared to PpHP1. The upper bound of the colour scale of the ‘MCR’ flux is set to 4

No remarkable differences in the fluxome between the two conditions were observed for the reference strain (Fig. 7A). In contrast, higher UG and PPP fluxes were observed at pH 3.5 than at pH 5 in strains PpHP1, PpHP6, PpHP8, PpHP15 (See Fig. 7B). In addition, a higher production of D-arabitol was also observed in most strains when growing at low pH (Fig. 7B and Additional Figure S2).

The fold-change of the relative fluxes of each recombinant strain compared to the reference strain (Fig. 7C) showed that the impact of each genetic modification in the flux of each strain followed a similar trend at pH 5 and pH 3.5 (Figs. 4 and 7B, respectively). 3-HP was produced at pH 3.5, but the yield was slightly lower than the one achieved at pH 5 for all the tested strains. For instance, the highest 3-HP producing strain at both pH values was PpHP8, which produced 3-HP at a yield of 0.084 ± 0.007 Cmol Cmol^− 1^ at pH 3.5 (0.081 ± 0.006 g g^− 1^), which is 23% lower than the product yield of the same strain at pH 5. See Additional Figure S3 for the comparison of the fluxes at pH 5 and 3.5 for this strain.

Regarding the NADPH production and consumption fluxes for each strain, the same trends were also observed at pH 3.5 (Additional Figure S4), that is, NADPH requirements in strain PpHP8 exceeded NADPH production from the PPP, meaning the flux through the cytosolic NADH kinase reaction compensated for that difference. In contrast, the NADPH production through the PPP in strain PpHP18 greatly exceeded the requirements. Moreover, the substrate uptake rate also followed the same trend for all the strains in both pH conditions (Additional Figure S4).

Altogether, these results contribute to the understanding of the adaptation of this yeast to a low pH at a fluxome level. Production of D-arabitol at acidic pH was increased for all the 3-HP-producing strains (from 2 to 20-fold). *P. pastoris* produces D-arabitol under several stress conditions, such as under hypoxia or osmotic stress [53, 55]. Thus, higher D-arabitol production at a lower pH is probably due to a stress response. Moreover, the biomass yield at pH 3.5 was lower for most strains (Fig. 7B), indicating a higher ATP requirement for maintenance. Such results have already been described in other yeasts grown at lower pH, such as *S. cerevisiae*, where the decrease in the biomass yield was also attributed to an increase in the ATP of maintenance [56]. Moreover, the 3-HP yield was lower than the one of the same strains at pH 5, consistently with previous studies describing 3-HP production in *S. cerevisiae* grown at pH 3.5 [57]. The observed decrease in the product yield when the ATP usage increases confirms that ATP is a limiting factor towards increasing the 3-HP yield. Similarly, increased D-arabitol production, which is a NADPH sink, can also explain the decrease in the 3-HP yield observed at low pH.

Still, as metabolic flux profiles at pH 3.5 remained mostly unchanged compared to those at pH 5, it is likely that strain engineering strategies at both pH will have the same outcome.

## Conclusions

This study describes the parallel characterization of a set of 3-HP-producing *P. pastoris* recombinant strains at two relevant process conditions (pH 5 and pH 3.5) using a HT approach that has allowed to save time and resources compared to conventional strain-by-strain sequential approach. Overall, we show, step-by-step, the setup of an optimized workflow for HT metabolic flux profiling of *Pichia pastoris*. It provides meaningful insights regarding the impact of each genetic perturbation on the metabolic flux distribution of the 3-HP producing strain, pointing to a competition for energy and carbon resources for either cell growth or 3-HP production as the major cause of the observed phenotypes, regardless of the pH of the culture. Thus, it is concluded that both acetyl-CoA and ATP limitations are the main bottlenecks hampering 3-HP production in *P. pastoris*. To overcome such bottlenecks, a strategy to increase the glycolytic fluxes (e.g., rewiring the regulatory mechanisms of this pathway and/or selecting cultivation conditions that favour higher glycolytic fluxes) should be addressed. Overall, this study will contribute towards the improvement of *P. pastoris* strains and bioprocess engineering strategies to produce 3-HP and other acetyl-CoA-derived products. Importantly, this study showcases the potential of automated fluoxomics workflows for accelerated strain characterisation in the context of the so-called Design-Build-Test-Learn cycle for metabolic engineering of industrially relevant microorganisms, beyond model organisms. To this end, we also provide an end-to-end description of the fluxomics workflow, as well as sharing the raw and processed datasets following the guidelines of good practices in publishing ^13^C-metabolic flux analyses studies, with the aim of contributing spreading the use of fluxomics analyses in *P. pastoris*.

## Electronic supplementary material

Below is the link to the electronic supplementary material.

**Additional Figure 1**. Flux maps of the parental P. pastoris strain (X-33) growing on methanol. Comparison of the flux map obtained in this study (batch mini bioreactor) with the previously reported flux maps in chemostat cultures (https://doi.org/10.1016/j.nbt.2019.01.005). **Additional Figure S2**. Bioprocess parameters of the parental P. pastoris strain and nine 3-HP-producing strains cultivated in glycerol batch mini bioreactor cultures at pH 3.5. **Additional Figure S3**. Flux map with the relative fluxes for the PpHP8 strain grown at pH 5 and pH 3.5. **Additional Figure S4**. Production and consumption rates of NADPH and specific glycerol uptake rates for each strain at pH 3.5.

## Data Availability

The datasets supporting the conclusions of this article are included within the article and its additional files. The Files can be downloaded from 10.34810/data673. The additional figures can be downloaded from the Electronic Supplementary materials.
