# Supplementary material for: High throughput 13C-metabolic flux analysis of 3-hydroxypropionic acid producing Pichia pastoris reveals limited availability of acetyl-CoA and ATP due to tight control of the glycolytic flux
Source: Microb Cell Fact. 2023 Jun 29;22:117. doi: 10.1186/s12934-023-02123-0 (PMC10308795; doi:10.1186/s12934-023-02123-0)
Supplement: Supplementary file 1 — Additional Figure 1. Flux maps of the parental P. pastoris strain (X-33) growing on methanol. Comparison of the flux map obtained in this study (batch mini bioreactor) with the previously reported flux maps in chemostat cultures (https://doi.org/10.1016/j.nbt.2019.01.005). Additional Figure S2. Bioprocess parameters of the parental P. pastoris strain and nine 3-HP-producing strains cultivated in glycerol batch mini bioreactor cultures at pH 3.5. Additional Figure S3. Flux map with the relative fluxes for the PpHP8 strain grown at pH 5 and pH 3.5. Additional Figure S4. Production and consumption rates of NADPH and specific glycerol uptake rates for each strain at pH 3.5. [file 12934_2023_2123_MOESM1_ESM.docx]

**Supplementary Figures**


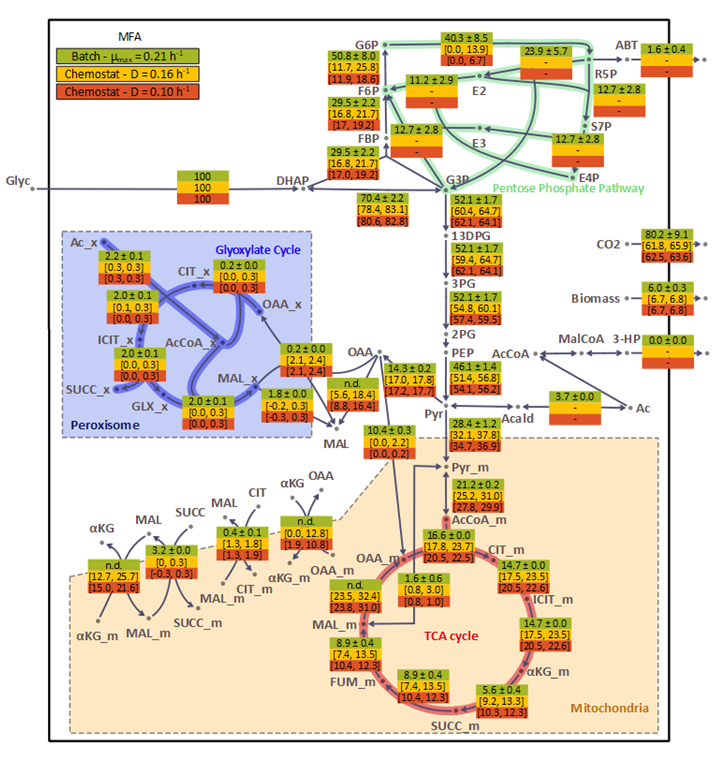


**Supplementary Figure 1.** Comparison of the flux maps of X-33 obtained in this study (green boxes), where the cells were grown in batch mode, and the results reported by Tomàs-Gamisans (2019), where the cells were grown in chemostat mode at D = 0.16 h^-1^ (yellow boxes) and
0.10 h^-1^ (red boxes). The abbreviation of the metabolites can be found on Figure 5.


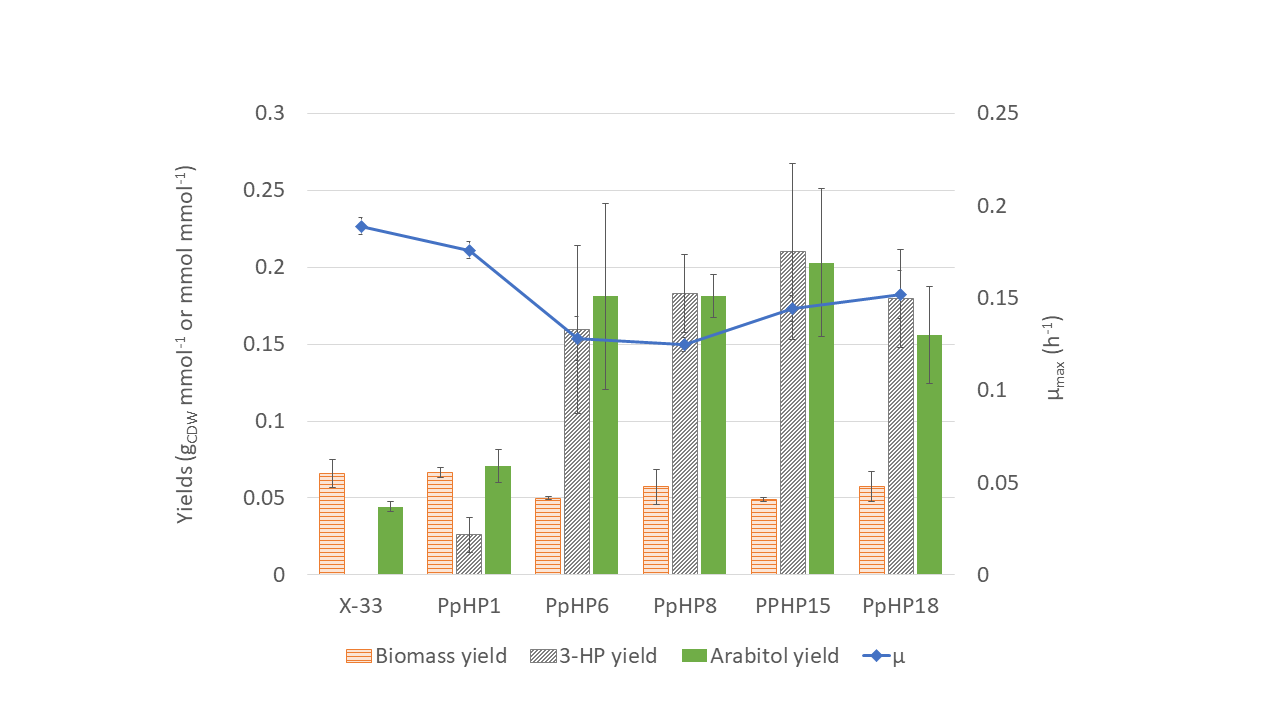


**Supplementary Figure S2.** Biomass, product, and by-product yields and µ_max_ of the parental *P. pastoris* strain and nine 3-HP-producing strains cultivated in glycerol batch mini bioreactor cultures at pH 5. Orange bars show the biomass yield, grey bars show the 3-HP yield, green bars show the arabitol yield, and blue diamond depict the µ_max_. Standard deviation of the replicates is depicted.


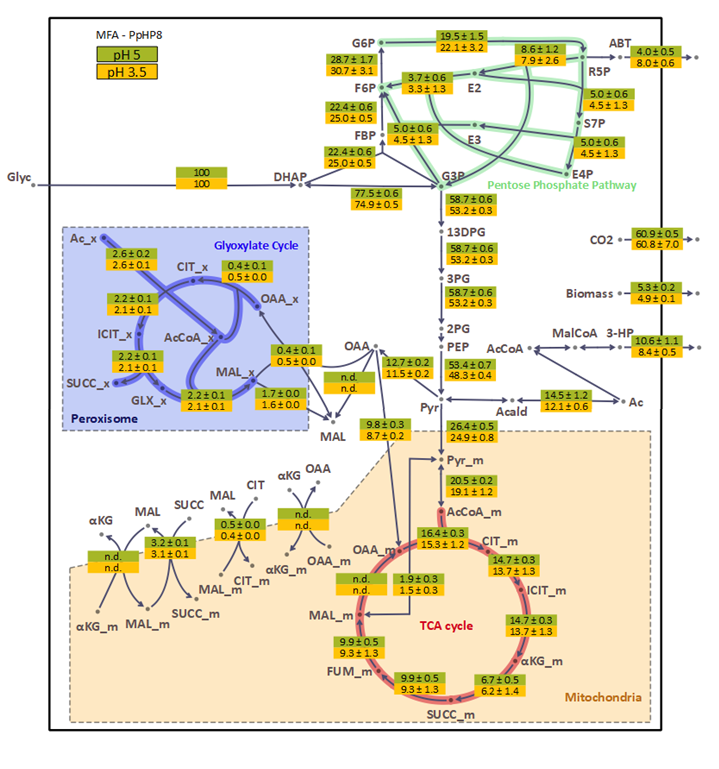


**Supplementary Figure S3.** Flux map with the relative fluxes for the PpHP8 strain grown at pH 5 and pH 3.5. Metabolite abbreviations can be found in the caption of Figure 5.


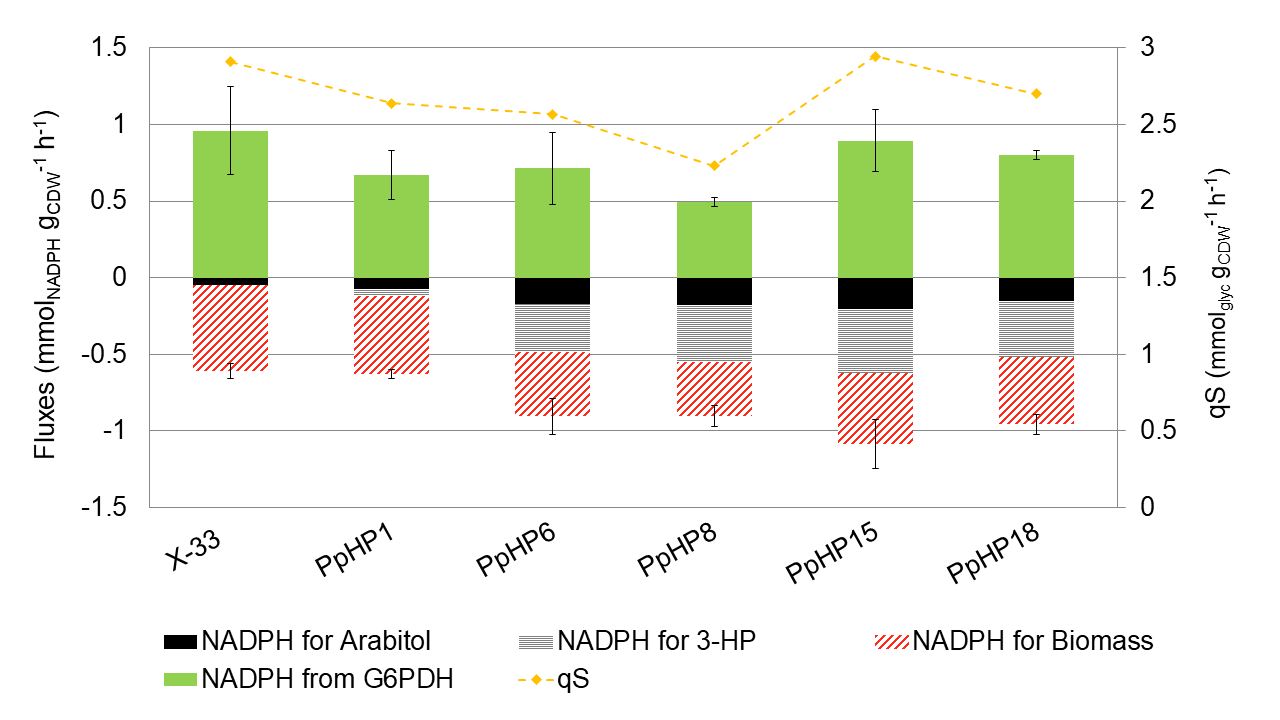


**Supplementary Figure S4.** Production and consumption rates of NADPH (estimated from the ^13^C-MFA results) and specific glycerol uptake rates for each strain.
